# Maximizing Acceleration and Change of Direction in Sport: A Case Series to Illustrate How the Force-Velocity Profile Provides Additional Information to That Derived from Linear Sprint Time

**DOI:** 10.3390/ijerph18116140

**Published:** 2021-06-07

**Authors:** Andrés Baena-Raya, Manuel A. Rodríguez-Pérez, Pedro Jiménez-Reyes, Alberto Soriano-Maldonado

**Affiliations:** 1 Department of Education, Faculty of Education Sciences, University of Almería, 04120 Almería, Spain; asoriano@ual.es; 2 SPORT Research Group (CTS-1024), CERNEP Research Center, University of Almería, 04120 Almería, Spain; 3 Center for Sports Studies, Rey Juan Carlos University, 28933 Madrid, Spain; peterjr49@hotmail.com

**Keywords:** acceleration, mechanical properties, explosive performance, assessment, team sports

## Abstract

Sprint running and change of direction (COD) present similar mechanical demands, involving an acceleration phase in which athletes need to produce and apply substantial horizontal external force. Assessing the mechanical properties underpinning individual sprint acceleration might add relevant information about COD performance in addition to that obtained through sprint time alone. The present technical report uses a case series of three athletes with nearly identical 20 m sprint times but with different mechanical properties and COD performances. This makes it possible to illustrate, for the first time, a potential rationale for why the sprint force-velocity (FV) profile (i.e., theoretical maximal force (F_0_), velocity (V_0_), maximal power output (P_max_), ratio of effective horizontal component (RF_peak_) and index of force application technique (D_RF_)) provides key information about COD performance (i.e., further to that derived from simple sprint time), which can be used to individualize training. This technical report provides practitioners with a justification to assess the FV profile in addition to sprint time when the aim is to enhance sprint acceleration and COD performance; practical interpretations and advice on how training interventions could be individualized based on the athletes’ differential sprint mechanical properties are also specified.

## 1. Introduction

The ability to efficiently perform a change of direction (COD), defined as rapidly accelerating, decelerating, and quickly changing speed and direction, greatly determines performance in the majority of sports [1,2]. Among other outcomes, it has been suggested that linear sprint, commonly assessed with photocells, might be a good indicator of individual sprint acceleration and maximal velocity capabilities during COD [1]. Previous research suggests that athletes who are faster in linear sprint tend to be also faster in COD [3,4]. However, considering only sprint time outcomes may overlook the mechanical capabilities underpinning individual sprint acceleration (i.e., the horizontal external force produced at various velocities during sprint running) [5], which might further explain both sprint acceleration and COD performance.

Exploring the influence of force production on COD performance has been a matter of research, especially through vertical force measures (i.e., maximal dynamic strength, eccentric strength and isometric strength) [2]. It has been demonstrated that stronger and more powerful athletes usually sprint and change direction faster than weaker individuals [6,7,8,9,10]. However, since horizontal force application is predominant during COD, the influence of horizontal force must also be assessed [11,12,13]. For example, Loturco et al. [3] reported that athletes with greater acceleration rates from 0–5 m had shorter COD times. Interestingly, Dos’Santos et al. [14] reported that athletes who were faster in the modified 505 COD test displayed greater horizontal to vertical peak (ES = −1.43) and mean braking force ratio (ES = −1.72) and greater horizontal to vertical peak and mean propulsive force ratio (ES = −2.67 and ES = −2.44, respectively) compared to their slower counterparts. Thus, the greater the horizontal GRF, the faster the transfer of force toward the new direction and, therefore, the faster the transition into the propulsive phase [14]. Although these findings clearly indicate that horizontal parameters underpin COD performance, athletes and practitioners do not usually have access to force platforms, which highlights the need to use alternative methods to evaluate horizontal force application during acceleration.

Samozino et al. [15] proposed the sprint force-velocity (FV) profile to describe athletes’ ability to specifically apply high amounts of GRF in the horizontal direction at various speeds during sprint acceleration, summarized through the maximal force (F_0_), velocity (V_0_) and power output (P_max_), the ratio of net horizontal resultant GRF (RF) and the index of its linear decrease as velocity increases (D_RF_) as derived exclusively from anthropometric and spatiotemporal data using accessible practical devices [16]. Baena-Raya et al. [12] recently reported that sprint FV profile parameters showed stronger associations with COD performance than vertical FV profile in tennis, soccer and basketball players. Specifically, F_0_ (r = −0.83; *p* < 0.001) and P_max_ (r = −0.79; *p* < 0.001) were the sprint FV profile parameters most strongly associated with COD performance in these three sports. In this line, Baena-Raya et al. also observed that higher F_0_, P_max_ and RF were strongly associated not only with higher COD performance but also with a reduced COD deficit in male and female basketball players [13]. Since acceleration capabilities play a key role in COD performance, the sprint FV profile represents an easily accessible tool that might provide coaches with unique information about the mechanical capabilities underpinning sprint acceleration performance, rather than relying solely on the final output (i.e., sprint time). Thus, assessing the sprint FV profile might add relevant information to allow for individual prescription of sprint acceleration training, which could in fact guide COD-specific training.

The aims of this communication are to (i) illustrate why the sprint FV profile provides key information about COD performance in addition to that derived from the sprint time alone, and (ii) provide practical interpretations and examples of how training interventions could be individualized based on the differential mechanical variables of athletes with different mechanical outputs.

## 2. Materials and Methods

This study is a descriptive analysis of 3 athletes (i.e., case series) with nearly identical 20 m sprint times but different acceleration mechanical variables and COD performance. The data used in the present study were collected during the abovementioned investigations [12,13]. We selected participants with nearly the same sprint time for the current communication. The FV profile as well as sprint and COD testing procedures were carried out on the same day. No familiarization session was included, because the testing procedures were part of the player’s in-season routine assessment. Prior to the tests, all participants performed a standardized warm-up protocol, including 5 min of jogging and 5 min of lower limb dynamic stretching. The specific warm-up consisted of 3 progressive sprints of 30 m at increased running velocities, before the sprinting test and 2 sub-maximal effort trials and 1 maximal effort trial before the COD tests. Athletes recovered for 3 min from the end of the specific warm-up to the beginning of every test. Participants gave their written consent before the initiation of the study. The study was approved by the local ethics committee (Ethical Application Ref: UAL-BIO2019/041), and all procedures were in accordance with the Code of Ethics of the World Medical Association (Declaration of Helsinki).

### 2.1. Procedures

#### 2.1.1. Sprint FV Profile Test

To determine the individual sprint FV profile, participants performed two maximal sprints of 30 m, with 4 min of recovery time between trials. All data were collected using a Stalker Acceleration Testing System (ATS) II radar device (Model: Stalker ATS II Version 5.0.2.1; Applied Concepts, Dallas, TX, USA). The radar device was attached to a tripod 10 m from the starting line at a height of 1 m, corresponding approximately to the height of participants’ centre of mass. The radar device sampled velocity-time data at 46.9 Hz. Participants initiated the sprint from a crouching position (staggered-stance). The velocity-time data were used to determine individual FV relationships (i.e., F_0_, V_0_, P_max_, RF and D_RF_) using inverse dynamic analysis applied to the body centre of mass, as validated by Samozino et al. [15]. The raw velocity data were fitted by a mono-exponential function using least-squares regression. The horizontal acceleration GRF was calculated from the changes in velocity over time, combined with the body mass and aerodynamic friction force [15]. Individual FV relationships were modelled to determine x-intercept and y-intercept (i.e., F_0_ and V_0_) and P_max_ (F_0_·V_0_/4). The sprint FV profile parameters were normalized to body mass. Furthermore, two pairs of photocells (Microgate, Bolzano, Italy) were positioned at the starting line and a distance of 20 m to measure sprint time at that distance during the same maximal linear sprint.

#### 2.1.2. Change of Direction Test

COD performance was assessed by the modified 505 test. Athletes began the test 0.3 m behind the pair of photocells placed on the starting line. The set of cones were set at 5 m from the start position. Athletes were instructed to accelerate as fast as possible along the 5 m distance, place their foot (dominant side) on the line, pivot and sprint through the finish (photocells placed at starting position) [17,18]. The reliability and validity of the modified 505 test for assessing COD ability during 180° turns have been previously reported [17,18]. Two trials were completed for each pivot foot, using the fastest time for analysis.

## 3. Results

**Figure 1:**
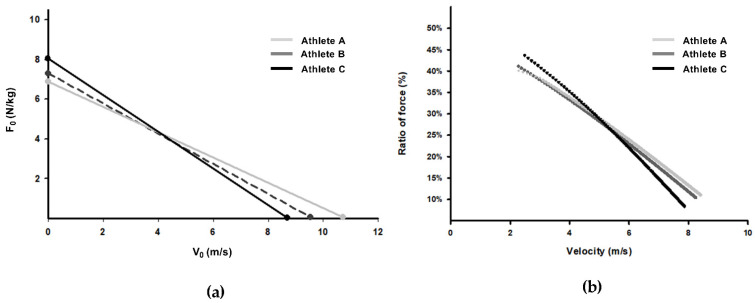
Sprint force-velocity profile mechanical variables from three different athletes with almost similar T_20 m time but different change of direction performance. (**a**) Sprint FV profiles (F_0_-V_0_ relationship) of three athletes; (**b**) Individual ratio of horizontal force application during linear sprinting. F_0_, theoretical maximal force; V_0_, theoretical maximal velocity; T_20 m, time in 20 m.

Table 1 shows the descriptive data of the sprint FV profile variables and performance in COD and sprint tasks from the three athletes. Figure 1 shows three different athletes, who cover a given distance (i.e., 20 m) in nearly the same time (A, 3.33 s; B, 3.37 s; C, 3.36 s), despite presenting different mechanical variables and COD performance. Athlete A presents higher V_0_ (10.6 m/s) compared with athlete B (9.72 m/s) and C (8.78 m/s) as well as more positive D_RF_ (−6.21% vs. −6.34% and −8.19%, respectively). In contrast to athlete A, athlete B presents slightly higher force capabilities and slightly lower V_0_ and D_RF_ values, despite presenting virtually the same 20 m sprint time. Finally, athlete C displays greater F_0_ (8.01 vs. 6.85 and 6.82 N/kg), RF_peak_ (50 vs. 47 and 43%) and P_max_ (17.47 vs. 16.53 and 17.4 W/kg) than athletes B and A, respectively, again leading to the same sprint time.

## 4. Discussion

This communication was designed to illustrate why the sprint FV profile provides key information about COD performance that might complement that derived from sprint time, through a case series of three athletes. We also aimed to provide practical interpretations and examples on how to individualize training to target the specific mechanical variables in different athletes. The main findings revealed faster COD time for athlete C, despite the lower 20 m sprint time in comparison with athletes A and B. Interestingly, athlete C displayed higher F_0_ and RF than athletes A and B during sprint acceleration, supporting the notion that high horizontal force application during both sprint acceleration and COD is important.

Sprint running and COD actions present similar mechanical demands during the acceleration phase. To cover a given distance in the shortest possible time, athletes need to produce and apply substantial horizontal external force at variable velocities [5,14,21,22]. Although it must be recognized that sprint time is a good indicator of COD performance [3,4], the sprint FV profile might still add unique information about the mechanical variables underlying sprint acceleration. In this regard, Baena-Raya et al. [12,13] suggested that for optimizing COD performance, not only the amount of force the athlete produces is important, but also how efficiently the force is transmitted onto the ground during sprint acceleration [23]. The present case series show how, despite all athletes presenting virtually the same 20 m sprint time, athlete C displayed the best performance in COD (2.34 s), followed by athletes B (2.41 s) and A (2.50 s). In this regard, the superior F_0_ and RF_peak_ values of athlete C (Figure 1) concur with faster COD time, despite lower effectiveness at increasing running speed. Although lower DRF and higher V_0_ values undoubtedly determine linear sprint performance [5,21], previous research suggest that these variables influence COD time to a lower extent than F_0_, RF_peak_ and P_max_ (i.e., the paramount parameters for short accelerations) [12,13]. This is widely applicable to many sports in which COD maneuvers take place in short distances.

**Figure 2:**
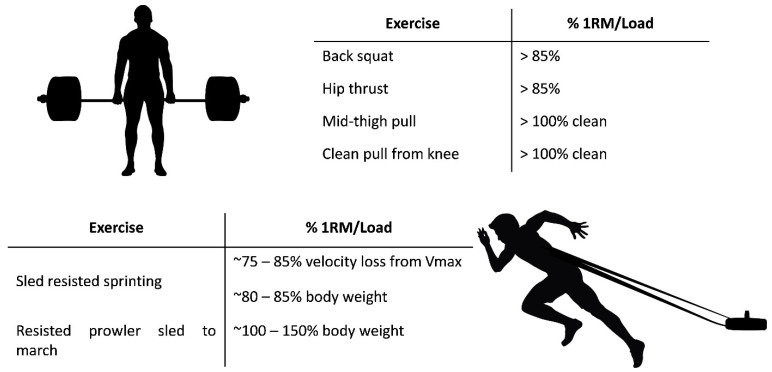
Suggested exercises to improve force production and mechanical effectiveness, adapted from Hicks et al. [24], Morin et al. [25] and Lahti et al. [26].

Coupling the sprint FV profile to COD actions may provide unique information for coaches to maximize acceleration capabilities through training interventions, which in turn may translate into improved COD performance. Previous studies conducted linear mixed models with 54 athletes (tennis, soccer and basketball players) to provide an estimation of the magnitude of association of FV profile variables with COD time in the whole sample, observing consistent association such that higher scores of F_0_, RF_peak_ and P_max_ were associated with lower COD [12]. Similarly, each unit change in these mechanical variables was associated with faster COD and reduced COD deficit in both male (n = 48) and female (n = 23) athletes [13]. From a practical perspective, strength and conditioning coaches may target specific areas of the FV profile through selected exercises to improve individual acceleration performance [24]. Thus, to improve short sprint acceleration, the training program should target F_0_ and RF_peak_ as a priority. Interestingly, there is consistent evidence supporting the use of force-oriented exercises to improve force production and mechanical effectiveness at low velocities (Figure 2) [24,25,26]. Specifically, heavy-load resistance exercises (i.e., >80% 1RM) are suggested to overcome inertia at the start of the sprint acceleration, whereas resisted sprint training (e.g., 0–10 m using either a maximal velocity reduction of 75–85% from V_max_ [26] or 80–85% body weight) or heavy sled pulls to march at >100% body weight would be advisable for increasing the ability to produce and apply force in the horizontal direction.

Of note, besides the potential usefulness of different resistance training programs to maximize acceleration capabilities and consequently COD performance, strength and conditioning coaches should also focus on training movement mechanics during COD not only to mitigate the athlete’s risk of injury but also to optimize the task-specific efficiency [11,27,28]. For further examples of exercises and strategies for this purpose, see recent investigations published by Dos’Santos et al. [11,27,28]. Although the abovementioned evidence suggests the potential influence of the sprint FV profile on COD performance, future research should assess whether maximizing the specific sprint FV profile mechanical variables (i.e., F_0_, RF_peak_) through an acceleration-based training program translates into improved COD performance.

## 5. Conclusions

This communication illustrates, through a case series of three athletes with nearly identical 20 m sprint times but different mechanical variables (i.e., F_0_ and RF_peak_) and COD performance, how the sprint FV profile provides unique information that might not be obtained through sprint times. Since acquiring this information has potential implications for individualized training prescription, we recommend practitioners assess the sprint FV profile to maximize acceleration capabilities, which in turn may result in improved COD performance.

## Figures and Tables

**Table 1:** Descriptive data of the sprint FV profile mechanical variables and performance in COD and sprint tasks from three different soccer athletes with respect to normative data of medium-level soccer players reported by Jiménez-Reyes et al. [19] and Haugen et al. [20].

	Athlete A	Athlete B	Athlete C	Normative Data
**Weight (kg)**	72.5	72.3	72.0	77.0 ± 8.00
**F_0_ (N/kg)**	6.82	6.85	8.01	6.73 ± 1.04
**RF_peak_ (%)**	43%	47%	50%	45.40 ± 1.20
**P_max_ (W/kg)**	17.47	16.53	17.47	14.90 ± 1.72
**V_0_ (m/s)**	10.06	9.72	8.78	8.89 ± 0.50
**D_RF_** **(%)**	−6.21%	−6.34%	−8.19%	8.40 ± 0.60
**T_20 m (s)**	3.33	3.37	3.36	3.44 ± 0.09
**Modified 505 COD (s)**	2.50	2.41	2.34	2.44 ± 0.11

F_0_, theoretical maximal force; RF_peak_, peak in the ratio of force; P_max_, maximal power output; V_0_, theoretical maximal velocity; D_RF_, index of force application technique; COD, change of direction.

## Data Availability

The data presented in this study are available on request from the corresponding author.
